# Evidence for arrogance: On the relative importance of expertise, outcome, and manner

**DOI:** 10.1371/journal.pone.0180420

**Published:** 2017-07-06

**Authors:** Maxim Milyavsky, Arie W. Kruglanski, Marina Chernikova, Noa Schori-Eyal

**Affiliations:** 1 Department of Psychology, University of Maryland, College Park, Maryland, United States of America; 2 Department of Psychology, Interdisciplinary Center, Herzliya, Israel; Mälardalen University, SWEDEN

## Abstract

Arrogant behavior is as old as human nature. Nonetheless, the factors that cause people to be perceived as arrogant have received very little research attention. In this paper, we focused on a typical manifestation of arrogance: dismissive behavior. In particular, we explored the conditions under which a person who dismissed advice would be perceived as arrogant. We examined two factors: the advisee’s competence, and the manner in which he or she dismissed the advice. The effect of the advisee’s competence was tested by manipulating two competence cues: relative expertise, and the outcome of the advice dismissal (i.e., whether the advisee was right or wrong). In six studies (*N* = 1304), participants made arrogance judgments about protagonists who dismissed the advice of another person while the advisees’ relative expertise (compared to the advisor), their eventual correctness, and the manner of their dismissal were manipulated in between-participant designs. Across various types of decisions and advisee-advisor relationships, the results show that less expert, less correct, and ruder advisees are perceived as more arrogant. We also find that outcome trumps expertise, and manner trumps both expertise and outcomes. In two additional studies (*N* = 101), we examined people’s naïve theories about the relative importance of the aforementioned arrogance cues. These studies showed that people overestimate the role of expertise information as compared to the role of interpersonal manner and outcomes. Thus, our results suggest that people may commit arrogant faux pas because they erroneously expect that their expertise will justify their dismissive behavior.

*“Through insolence comes nothing but strife*, *But wisdom is with those who receive counsel*.*”–*Proverbs 13:10

*"For by the grace given to me I say to everyone among you not to think of himself more highly than he ought to think"—*Romans, 12:3

“*I am not arrogant*, *I am just better than you*”—Internet

## Introduction

Arrogance is one of the most unpleasant and ineradicable manifestations of human nature—one that has been condemned since ancient times. Buddhists identified *māna* (arrogance) as one of the five poisons of the mind in the Mahayana, and as one of the ten fetters in the Theravada tradition [1,2]. Ancient Greeks had the notion of *hubris*, which referred to actions that humiliated the victim for the pleasure or gratification of the abuser. As the quotes above show, arrogance was also viewed as a sin in the Abrahamic religions. Contemporary research confirms these early insights showing that arrogant people are less liked by others, and perceived as less sociable, less intelligent, and less productive [3–6].

Despite its bad reputation, arrogance remains fairly common to this day. An online survey we conducted on the Amazon’s Mechanical Turk (*N* = 335) showed that as many as 84% of the respondents reported encountering arrogant behavior at least once a month, and as many as 46% of the respondents admitted behaving arrogantly themselves (data in S1 Dataset.). Evidently, arrogant behavior is a frequent social occurrence. But why do people keep behaving arrogantly?

One surprising answer to this question may be that certain behaviors are perceived as arrogant even though they were not intended as such by the actors. In other words, arrogant faux pas may be due to individuals’ misconceptions of the conditions under which their behavior will be perceived as arrogant. An interesting manifestation of arrogance in this regard is dismissive behavior, since people may erroneously think that their dismissal of others will be justified by their actual superiority in competence.

People may behave dismissively towards others in various ways. In some cases, individuals may dismiss others passively, for example, by not looking at them [7,8], by not paying attention to what their partner feels, thinks and says [9–11], or by not answering them [12]; in other cases, the dismissal may be more active, such as demeaning others [13] or interrupting them [14]. In this paper we focus on one of the most studied forms of dismissive behavior: the dismissal of advice [15]. Indeed, research shows that people tend to dismiss others’ advice in various social contexts, such as close and work relationships and on various topics, such as matters of fact and matters of taste, even when such behavior is not rationally justified [16–22].

Clearly though, not every dismissal of advice signals arrogance. It may indicate other traits, attitudes or mental states of the advisee such as independence, unfriendliness or self-confidence. The purpose of our paper is to examine the circumstances under which the dismissal of advice will appear arrogant, and to understand whether people have adequate expectations as to how such dismissals will be perceived.

### The determinants of perceived arrogance

Johnson and his colleagues [6] defined arrogance as "a set of behaviors that communicates a person’s exaggerated sense of superiority, which is often accomplished by disparaging others". Thus, when people encounter a dismissal of advice, they will consider it arrogant if they attribute it to the advisee’s desire to establish his or her superiority over the advisor. What can lead to such an attribution?

The literature on person perception identifies two main factors that determine people’s perception of other individuals: agentic competence and interpersonal behavior [23–26]. Both of these factors seem to be highly relevant for the perception of dismissive behavior. Namely, whether the dismissal of advice will appear arrogant may depend on the interpersonal manner of the dismissal, and on the extent to which the dismissal can be justified by the advisee’s competence. We explain below how the advisee’s manner and competence may influence whether his dismissal of advice will be perceived as arrogant. We also theorize about the relative importance of these factors—namely, which of them should have a greater impact on arrogance judgments.

#### Interpersonal manner

The most direct and obvious indicator of arrogance is the manner of the dismissal. If the very manner of a dismissal is disparaging, it will be difficult to explain it as anything except a desire to establish one’s superiority. Even though previous studies on the perception of arrogance did not manipulate the interpersonal manner of the target person directly, their findings are consistent with this prediction. For example, Hareli and his colleagues [27] showed that people who attributed their success to their ability were perceived as more arrogant than those who attributed it to other reasons such as effort, luck or the help of others. Importantly, people’s attribution of success to their own ability was perceived as an attempt to establish their superiority over others. Thus, when a person described her success in a manner that implied her steady superiority, participants concluded that she was more arrogant.

#### Competence

The advisee’s lower (compared to the advisor) competence may also point to arrogance as the underlying motive of her advice dismissal. In particular, if an advisee unjustifiably rejects the advice, people may suspect that this behavior is motivated by her desire to establish her superiority over the adviser, or her unwillingness to recognize the adviser’s superior (or equal) competence. Conversely, if the dismissal of advice can be justified by the advisee’s higher competence, the arrogance explanation should seem less likely.

Previous research on the perception of arrogance is consistent with this reasoning. Some indirect support for this idea comes from the research of Johnson and his colleagues [6]. More specifically, the researchers asked employees to rate their co-workers’ arrogance and performance at work, and also measured the co-workers’ intelligence. The results showed that less intelligent and poorer performing co-workers were rated as more arrogant. That is, presumably, co-workers whose behavior could *not* be justified by their competence were perceived as more arrogant. Stronger evidence comes from experimental studies conducted by Hareli and his colleagues [28]. The authors found that when the success of a person who boasted was explicitly justified by his actual ability, he was perceived as less arrogant.

### Present research

Previous research on the perception of arrogance focused on one type of arrogant behavior: boasting [27–30]. In this paper, we turn our attention to the study of another, presumably more typical, manifestation of arrogance: dismissive behavior. More specifically, we consider situations that are quite common in personal and professional life: times when a person offers advice to an interaction partner, and the advisee dismisses it.

We conducted a preliminary study to examine our assumption that dismissive behavior is a typical manifestation of arrogance. In an online survey conducted on Amazon’s Mechanical Turk (*N* = 256), we asked participants to name behavioral cues that make them think that other people behave arrogantly. We then coded the frequency of participants mentioning *dismissive* and *boasting* behaviors in their answers. The results showed that dismissive behavior was mentioned in 58% of respondents’ answers, while boasting was mentioned in only 38% of answers (data in S2 Dataset.). Thus, dismissive behavior seems to be a more prototypical case of arrogant behavior than boasting.

Based on the literature reviewed above, we hypothesized that the perception of an advice dismissal will depend on the relative competence of the advisee and on the manner of the dismissal. Below, we outline our hypotheses about competence, interpersonal manner, and their relative importance for arrogance judgments.

Previous research on advice-taking explored various cues that people use to infer competence [31,32]. In this paper, we focused on two of them: *expertise* and the *outcome* of the decision. In what follows, we explain their importance for and influence on the perception of arrogance.

#### Expertise

From early childhood human beings are attuned to others’ expertise [31–39]. “Says who?” is what we immediately think when exposed to someone else’s opinion [40]. Indeed, it makes more sense to listen to someone who has a reputation for epistemic authority than to someone who lacks it. Research shows that decision makers tend to assign higher weight to the opinions of experts [36–38]. People constantly consult with others whose social status, profession, education, experience or other cues signal their greater or lower expertise [15]. Oftentimes, however, advisees are tempted to stick to their own opinions and to discount or dismiss those of others [17,39,41,42]. It is important to know, then, what social costs they may incur for such a behavior. More specifically, will their *lower* expertise put them at greater risk of being judged as arrogant, and can their *higher* expertise protect them from this danger?

As was explained above, dismissing the advice of a person with greater expertise may seem less justifiable and make an arrogant motive seem more likely. We thus hypothesized that *the perceived arrogance of a person who dismissed advice would be inversely related to his/her expertise relative to the advisor*. *That is*, *a more expert advisee would be perceived as less arrogant*, *and a less expert advisee would be perceived as more arrogant* (Hypothesis 1).

#### Outcomes

The outcome of a behavior is often viewed as the best criterion of its worthiness: if it’s good, the behavior was good; if it’s bad, something was wrong with the behavior. This notion lies at the core of numerous philosophical theories of ethics, such as consequentialism and utilitarianism [43,44]. People may use the same heuristic with respect to the outcome of the dismissal of advice. Namely, observers may interpret the advisee’s eventual correctness as evidence of his or her better knowledge, thus undermining the “arrogance” interpretation. Conversely, an advisee’s mistake may be perceived as an indicator of poorer knowledge and thus function as an aggravating circumstance that leads to higher ratings of his arrogance.

Although decision analysts have criticized the consequentialism approach, pointing out that good decisions can lead to bad outcomes (and vice versa) [45,46], the literature on hindsight and outcome biases has shown that people cannot ignore the outcome information while judging the *a priori* likelihood of the outcome, even when they are explicitly instructed to do so [47–49]. More relevant to our case, the outcome bias was found when people were making judgments of other people’s behavior. Specifically, Gino and her colleagues [48] found that the outcomes of unethical behaviors influenced people’s judgments of the ethicality of these behaviors. Moreover, people may even use the outcome of the advice dismissal intentionally in their arrogance judgments, because as was mentioned above, they may treat it as evidence of the advisee’s competence in that particular issue. Based on these considerations, we hypothesized that *perceived arrogance would be inversely related to the decision’s outcome*. *That is*, *an outcome confirming the advisee’s opinion would make him appear less arrogant*, *while an outcome disproving the advisee’s opinion would make him appear more arrogant* (Hypothesis 2).

#### Expertise vs. outcomes

In addition to investigating the effects of expertise and outcome separately, we were also interested in the relative importance of the two factors for the perception of arrogance. This question is interesting for two reasons. First, if indeed the less expert advisee's dismissal of advice makes her look arrogant, the question arises whether she could be rehabilitated by her eventual rightness. And vice versa: should a more expert person be afraid that in the case of an erroneous dismissal of advice, she will be perceived as arrogant?

At first glance, it seems obvious that expertise should eclipse the outcome of a single decision, because expertise is based on one’s repeated experiences and extensive learning, while one decision may be right or wrong through mere accident [46]. On the other hand, an outcome may seem like a more valid cue of competence on any given issue. For example, a cook may know about cooking in general, but may not know which specific dough is best for a particular cake. Indeed, the literature on heuristics and biases shows that people tend to neglect statistical information in favor of individual cases [50–52]. Based on this literature, we hypothesized that *outcome information should trump the effect of expertise on arrogance judgments*. *More specifically*, *a positive outcome should decrease the aggravating effect of the advisee’s lower expertise on his perceived arrogance*, *and a negative outcome should decrease the mitigating effect of the advisee’s higher expertise* (Hypothesis 3).

#### Interpersonal manner

The hypothesis about the influence of the interpersonal manner on the perception of arrogance follows directly from the aforementioned definition of arrogance [6]. Although it has received partial support with respect to boasting behavior [27,28], it must be noted that attributing one’s success to own ability (vs. effort, luck, and the help of others) and celebrating one’s success excessively [29] are relatively innocuous behaviors that do not disparage anyone directly. In our studies, we wanted to examine how people would perceive a behavior that is specifically directed toward another person. Importantly, unlike previous researchers, we examined dismissive rather than boasting behavior. We expected that *a rude (vs*. *neutral or polite) dismissal of advice would be perceived as more arrogant* (Hypothesis 4).

#### Interpersonal manner vs. competence

Another question we were interested in is which of the two factors—interpersonal manner or competence—is more important for arrogance judgments. This question is of both theoretical and practical importance. On the theoretical side, the answer to this question could shed light on the nature of arrogance. If arrogance is primarily an intellectual phenomenon wherein people unjustifiably dismiss others’ opinions [53,54] then competence considerations should trump considerations of interpersonal manner. However, if arrogance is primarily an interpersonal phenomenon wherein one is trying to establish his or her superiority over others, then considerations of interpersonal manner should prevail.

On the practical side, this question is of interest because sometimes more competent people (e.g. parents, teachers, or managers) might be tempted to dismiss the opinion of their less competent counterparts (e.g., children, students, or junior colleagues) in a disrespectful manner, assuming that their expertise or eventual correctness will justify their behavior. For example, Johnson and his colleagues [6] argued that Muhammed Ali's claims of being "the greatest" were not at all arrogant since he was actually winning every fight. But is this assumption correct?

Previous research on social perception suggests that interpersonal considerations should trump considerations of competence [23–25]. For example, Abele and Wojciszke [55] found that interpersonal content explained substantially more variance in personality traits than competence content. In addition, people rated interpersonal (vs. competence) traits as considerably more important for evaluating others. If so, we should expect information about the advisee’s interpersonal manner to eclipse information about his or her relative competence. This prediction should be especially true with regard to a disrespectful manner, since previous research has shown that negative information is perceived as more diagnostic of an actor’s true character than positive information [56–58]. Based on this literature, we predicted that *a rude (vs*. *neutral or polite) dismissal of advice would be perceived as more arrogant*, *regardless of the advisee’s relative expertise* (Hypothesis 5) and *the outcome of the dismissal* (Hypothesis 6).

The last issue we explored in this research was related to people’s naïve theories about the relative importance of the aforementioned factors for arrogance judgments. In particular, we wanted to check whether people’s naïve theories corresponded to our findings on the relative importance of expertise, outcomes, and interpersonal manner for perceptions of arrogant behavior. Answering this question would allow us to assess the triviality (or non-triviality) of our findings. In particular, we predicted that although outcome information trumps expertise information when people judge an advisee’s dismissal of advice, *people would rate expertise as more important for arrogance judgments when asked about it hypothetically* (Hypothesis 7).

In addition, as mentioned in the beginning of the paper, inaccurate expectations of when competence and interpersonal manner would make a dismissive behavior appear arrogant could explain why people behave arrogantly. More specifically, if people expected expertise to be as important as manner for the perception of arrogance, they might mistakenly assume that their rude dismissals of others’ opinions would be excused by their greater expertise. In line with this reasoning, we predicted that although manner information trumps expertise information when people judge an advisee’s dismissal of advice, *people would not rate manner as more important for arrogance judgments when asked about it hypothetically* (Hypothesis 8).

### Overview of the studies

We used vignettes to test our hypotheses, as this method is commonly used in the literature on person perception[27–30]. In particular, participants were asked to rate the arrogance of an advisee who dismissed the advice given to him or her by another person. The vignettes were constructed such that the advice would be useful for the advisee, so its dismissal would not seem justified from a rational point of view [59]. Namely, the advisor always suggested improving the advisee’s decision. Also, in heeding advice, the advisee did not have to completely give up on his or her opinion. Rather, he or she could always adjust his or her prior decision to the advice to some extent. For example, in one vignette, an advisor suggested to an advisee working on the development of a new website that he should make the colors of the website darker. Thus, participants could always assume that the categorical dismissal of advice reflected the advisee’s arrogance, and the question was just the extent to which it did.

We tried to investigate typical advice situations, such as friendly and professional advice, advice about matters of fact and matters of taste, and advice about more and less important decisions. Since it was not the goal of the present research to examine the effects of these factors, we did not manipulate them systematically; rather, we just wanted to show that our conclusions about the perception of dismissive behavior hold across various types of advisee-advisor relationships and decisions.

In a series of six experiments, we examined how the advisee’s relative expertise, the outcome of the advice dismissal, and its manner influenced how arrogantly the advisee was perceived. Importantly, in all experiments, the factors of interest were manipulated in a between-participants design in order to preclude any effects of participants’ naïve theories about how these factors should influence perceptions of arrogance. In particular, in Study 1, we manipulated the advisee’s and the advisor’s expertise and asked participants to rate the advisee’s arrogance. In Study 2, we manipulated the outcome of the advice dismissal so that it would indicate the advisee being either correct or incorrect. In Study 3a, we manipulated the advisee’s expertise and the outcome of the dismissal orthogonally, to examine which of those factors influences the perception of arrogance more. Study 3b was conducted in order to replicate the results of the previous three studies using a different method of measuring the perception of arrogance. Specifically, instead of asking participants to rate the advisee’s arrogance, we asked them to select the most appropriate label for his or her behavior (e.g., arrogant, confident, or independent). In addition, we wanted to clarify whether a negative (and not just a positive) outcome can moderate the effect of expertise. In Study 4, we manipulated the advisee’s relative expertise and the manner of his or her advice dismissal, in order to examine which of these two factors influences the arrogance judgments more. In Study 5, we aimed to replicate the interaction pattern obtained in Study 4, indicating the greater importance of manner, with another cue of competence: the outcome of the advice dismissal. In addition to these six experiments, we conducted two more studies in which we asked participants to hypothetically rate the importance of the factors of interest for their perception of arrogance.

## Study 1: Expertise

The purpose of Study 1 was to examine whether the advisee’s greater expertise would attenuate his or her perceived arrogance. According to our Hypothesis 1, the dismissal of another person’s advice by the advisee should be judged as less arrogant when the advisee has greater expertise than the individual offering advice.

### Method

#### Participants

One hundred and nineteen participants (70 females), ranging in age from 20 to above 40, took part in the study on Amazon’s Mechanical Turk for 20 cents (USD) each. Across all studies, there were participants from 52 countries. However, most of the sample in each study was from the US (Study 1–82.4%, Study 2–92.7%, Study 3a – 90.1%, Study 3b – 90.1%, Study 4–91.7%, Study 5–96.7%, Study 6a – 88.0%, Study 6b – 90.2%).

#### Ethics statement

This research was approved by the Institutional Review Board of the University of Maryland. In all studies, participants conducted online surveys anonymously, so there was no risk of a confidentiality breach. The surveys involved reading a vignette about everyday life interpersonal situations, and evaluating the actors’ behaviors. So, there was no potential psychological discomfort in performing the studies. Before starting the surveys, the participants were given a written informed consent form. The consent form (approved by the IRB) described the procedures, the potential risks (none), the benefits and the payments in the study. The participants started answering the surveys only after having electronically signed the consent form.

#### Procedure and design

In order to examine whether arrogance judgments are influenced by the advisee’s *relative* expertise, we included three between-participants conditions. In Condition 1, the advisee was described as incompetent, and the advisor as competent; in Condition 2, both were described as competent, and in Condition 3, the advisee was described as competent and the advisor as incompetent. The logic of this design is as follows: higher arrogance ratings in Condition 1 versus Condition 2 could be attributed either to the *advisee's* relative or absolute incompetence. In other words, one could argue that the behavior of the advisee was considered more arrogant because he was incompetent in absolute terms and not because he was *less* competent than the advisor. However, such an explanation would not hold if the advisee’s behavior were judged more arrogant in Condition 2 than in Condition 3, since in both conditions the advisee is described as competent. Similarly, higher arrogance ratings in Condition 2 versus Condition 3 could be attributed either to the advisor’s relative or absolute incompetence. However, such an explanation would not hold if the same difference would be found between the Conditions 1 and 2 since in both he is described as competent. Thus, the relative expertise hypothesis would be supported *only* if we found higher ratings of arrogance in Condition 1 than in Condition 2, and in Condition 2 than Condition 3. Participants were presented with one of the following vignettes in a between-participants design:

***Condition 1***. Advisee: incompetent; Advisor: competent;
  Jamie has entered a robotics contest in the community; **he does not know very much about robotics**. Jamie is constructing a robot at home when a friend, Taylor, who **knows a lot about robotics**, comes to visit. Taylor looks at the robot and advises Jamie to upgrade the power supply. Jamie says that the power supply is perfect and no upgrading is needed.
***Condition 2***. Advisee: competent; Advisor: competent;
  Jamie has entered a robotics contest in the community; he **knows a lot about robotics**. Jamie is constructing a robot at home when a friend, Taylor, who also **knows a lot about robotics**, comes to visit. Taylor looks at the robot and advises Jamie to upgrade the power supply. Jamie says that the power supply is perfect and no upgrading is needed.
***Condition 3***. Advisee: competent; Advisor: incompetent;
  Jamie has entered a robotics contest in the community; he **knows a lot about robotics**. Jamie is constructing a robot at home when a friend, Taylor, who **does not know very much about robotics**, comes to visit. Taylor looks at the robot and advises Jamie to upgrade the power supply. Jamie says that the power supply is perfect and no upgrading is needed.

The participants were only asked to rate the extent to which Jamie’s behavior in each vignette was arrogant (1 –*not at all* to 7 –*very much*).

### Results

There were no significant effects of gender, so it will not be discussed further. An ANOVA with Expertise as a between-participants’ factor was run on the ratings of the advisee’s arrogance. This analysis also showed a significant effect of Expertise, *F*(2, 116) = 13.33, *p* < .0001, $\eta_{p}^{2}=.19$. The planned contrasts showed that the advisee’s behavior was judged as *more* arrogant when he had *lower* expertise (M = 4.87, SE = .28) than when he had *equal* expertise to that of the advisor (M = 4.00, SE = .26), *p* = .023, *d* = 0.53. Also, the advisee’s behavior was judged as *more* arrogant when he had *equal* expertise than when he had *higher* expertise than the advisor (M = 2.87, SE = .28), *p* = .003, *d* = 0.68 (data in S3 Dataset.).

### Discussion

As expected, the results of Study 1 showed that the *lower* the advisee’s relative expertise, the *more* arrogant his dismissal of the advisor’s opinion was perceived. These results support our Hypothesis 1, showing that dismissive behavior can be justified by one's actual superiority in competence. Can this conclusion be generalized to other cues of competence? We addressed this question in the Study 2 by manipulating the outcome of the advice dismissal.

## Study 2: Outcomes

The goal of Study 2 was to generalize the effect of competence to another potentially relevant cue of competence: the outcome of the advice dismissal. Namely, we aimed to examine Hypothesis 2, which states that an outcome confirming the correctness of the advisee’s opinion would make the dismissal of advice appear less arrogant, while an outcome confirming the advisor’s opinion would make the dismissal of advice appear more arrogant.

We also sought to generalize our conclusions about the effect of relative competence on the perception of arrogance from decisions on matters of fact to decisions on matters of taste, as previous research has shown that combining opinions can be beneficial for the latter type of decisions as well [21,60]. In order to do that, we added a new vignette in which advice about cooking was offered.

### Method

#### Participants

Two hundred and four participants (119 females), ranging from 20 to above 40 in age, took part in the study on Amazon’s Mechanical Turk for 20 cents (USD) each.

#### Procedure and design

Participants were presented with one of two vignettes: the Robotics vignette used in Study 1, or a new vignette in which a protagonist dismissed another person’s advice about cooking. The outcome of the dismissive behavior was also manipulated between-participants by describing the result of the advisee’s behavior as either a success or a failure; in the control condition, no outcome was mentioned (see below), yielding a Vignette (Robotics / Cooking) x Outcome (positive / negative / no information about the outcome) between-participants design:

***Cooking vignette***. Emily is a second year student in culinary school and has entered a pastry contest in her community. She is preparing a cake at her house when her friend Michael, who is a student in the same class, comes to visit. Michael tastes the dough and advises Emily to add more sugar to it. Emily says that the dough is perfect and no more sugar is needed. [**Negative outcome**: When the judges evaluate her cake during the contest, they give her a low mark arguing that the cake is not sweet enough; **Positive outcome**: When the judges evaluate her cake during the contest, they award her first place and say it is perfect; **Control**: no information about outcome]

***Robotics vignette*.** Jamie has entered a robotics contest in the community. Jamie is constructing a robot at home when a friend, Taylor, comes to visit. Taylor looks at the robot and advises Jamie to upgrade the power supply. Jamie says that the power supply is perfect and no upgrading is needed. [**Negative outcome**: When the judges evaluate Jamie's robot during the contest, they give it a low mark arguing that the power supply does not fit the robot; **Positive outcome**: When the judges evaluate Jamie's robot during the contest, they award it first place and say it is perfect; **Control**: no information about outcome]

Participants were asked to rate the extent to which the advisee's behavior (Emily's or Jamie's) was arrogant, and the extent to which both the advisee and the advisor were competent (1—*not at all* to 7—*very much*) in the relevant domain (i.e., cooking or robotics). The perceived competence of the actors in the vignette was measured as a potential mediator.

### Results

There were no significant effects of gender, so it will not be discussed further. An ANOVA with Outcome and Vignette as between participants’ factors was run on the ratings of the advisee’s arrogance. This analysis showed a significant effect of Outcome, *F*(2, 198) = 16.20, *p* < .0001, $\eta_{p}^{2}$ = .14 The planned pair-wise comparisons showed that compared to the control condition (M = 3.90, SE = .20), the advisee's behavior was judged as *more* arrogant when he or she turned out to be wrong (M = 4.51, SE = .20), *p* = .033, and as *less* arrogant when he or she turned out to be right (M = 2.91, SE = .20), *p* = .001. No other effects were significant, *p* > .24.

Thus, supporting Hypothesis 2, the outcome of a person’s dismissive behavior can be perceived as an indicator of his or her relative competence, and thereby signal arrogance. In order to test whether the effect of outcomes on arrogance ratings was mediated by the advisee's relative competence, we calculated a measure of the advisee's relative competence by subtracting participants' ratings of the advisor’s competence from those of the advisee. Lower scores on this measure denoted lower levels of the advisee's relative competence. The outcome conditions were coded in an ascending order (-1 for negative outcome, 0 for the control condition and +1 for the positive outcome).

We used model 4 in PROCESS [61] to estimate the indirect effect of outcomes on arrogance ratings through the advisee’s relative competence. The outcome manipulation predicted the advisee's relative competence, *β* = .62, *p* < .0001, that is, the more positive the outcome of the advisee's behavior, the more competent he was judged to be as compared to the advisor. The advisee's relative competence had a negative effect on the arrogance judgments, *β* = -.41, *p* < .0001, that is, the less competent the advisee was perceived to be compared to the advisor, the more his or her behavior was judged as arrogant. Consequently, the indirect effect of the outcome manipulation through the advisee's relative competence on the arrogance judgments estimated with 20,000 bootstrapped samples was significant, *β* = -0.18, 95% CI [-.29, -.08] (data in S4 Dataset.).

### Discussion

Across two types of decisions—factual and hedonic—analyses showed that outcome considerations affected arrogance judgments to a substantial extent. In particular, negative outcomes of dismissive behavior made it appear more arrogant, while positive outcomes made it appear less arrogant. This effect was mediated by the advisee’s perceived relative competence. That is, more positive outcomes of the advisee's behavior made him or her look more competent (relative to the advisor) and thereby made his or her behavior look less arrogant. These findings complement the results of Study 1 and provide support for Hypothesis 2, since they show that another cue of competence, the outcome of the advice, dismissal can justify dismissive behavior. However, which of these two cues of competence—expertise or outcome—is more important for arrogance judgments? We addressed this question in the next two studies.

## Study 3a: Outcomes vs. expertise

Studies 1 and 2 established the effects of expertise and outcomes on arrogance judgments. The main goal of Study 3a was to examine our Hypothesis 3 that the outcome of the advice dismissal would moderate the effect of the advisee’s expertise on arrogance judgments. Although, rationally, expertise considerations should prevail over outcomes, since outcomes are not known when the behavior is carried out, Study 2 showed that the outcome of the dismissal has a large effect on arrogance judgments $\left(\eta_{p}^{2}=.14\right)$. Presumably, this effect was so large because participants interpreted the outcome information as a direct cue of the advisee’s competence (or incompetence) in the particular issue. If so, the outcome might seem more indicative of a particular assertion than the advisee’s expertise. We expected participants to apply the following logic in their arrogance judgments: if a person turned out to be right, it is likely that he knew what he was talking about and therefore his relative expertise should not matter. If the person was wrong, it is likely that he did not know what he was talking about. In that case, dismissing the expert’s advice appears less justifiable and therefore more arrogant than dismissing the novice’s advice. In order to test these predictions, we manipulated both outcome and expertise information orthogonally.

A secondary goal of this study was to extend the external validity of the effects of expertise and outcomes found in Studies 1 and 2 (Hypotheses 1 & 2) to a work context. In order to do that, we added a new vignette in which a web designer dismissed his or her colleague’s advice about the design of a website.

### Method

#### Participants

Two hundred sixty four participants (142 females), ranging from 18 to 79 in age (Mean = 34.39, SD = 11.94), took part in the study on Amazon’s Mechanical Turk for 20 cents (USD) each. Two hundred thirty seven participants were from the United States, and the rest were from other countries.

#### Procedure and design

As in the previous studies, participants were presented with a situation in which a protagonist dismissed potentially valuable advice outright. This time we manipulated the advisee’s superiority, both in terms of his or her relative expertise and of the outcome of his or her dismissal of the received advice. The advisee’s relative expertise was manipulated by describing the advisor either as a novice or as an expert. The outcome was manipulated in the same way as in Study 3a.

In order to extend the generality of our conclusions from the context of friendly relationships between the advisee and the advisor to the context of working relationships, we added a vignette describing professional advice (i.e., the web-design vignette). Consequently, the design of the present study was 2 Type of Relationships (friendship / work) x 2 Outcome (positive / negative) x 2 Advisee’s Relative Expertise (lower / higher). Each of these three factors was manipulated between participants; that is, each participant was presented with only one version of the following 8 vignettes:

***Cooking vignette*.** Emily is a second year student in a culinary school and has entered a pastry contest in her community. She is preparing a cake at her house when her friend Michael, who **works as a professional chef** / **is going to start his studies at the same culinary school**, comes to visit. Michael tastes the dough and advises Emily to add more sugar to it. Emily says that the dough is perfect and no more sugar is needed. When the judges evaluate her cake during the contest, they **award her first place and say it is perfect** / **give her a low mark arguing that the cake is not sweet enough**.
***Web-design vignette*.** Alex has been working as a web designer in a computer company for a few years now. He is working on a new website and has to present it to the customers next week. While Alex is working on the website, his colleague Steve, who has been working as a web designer in the company for **twenty years / several months**, enters the room. Steve looks at the website and comments that the colors of the website are too bright and suggests that Alex should make them darker. Alex says that the colors are perfect and nothing should be changed. Next week, when the customers look at the website, they say that **the website is perfect / the colors on the website are too bright**.

After reading the vignette, participants were asked to rate the extent to which the advisee's (Emily's or Alex's) behavior was arrogant and confident (1—*not at all* to 7—*very much*). The confidence item was added to establish the discriminant validity of the arrogance measure.

### Results

#### Arrogance judgments

An ANOVA with Type of Relationships, Outcome, and Advisee’s Relative Expertise as between-participants factors was conducted on the ratings of the advisee’s arrogance. The results revealed a significant effect of Outcome, *F*(1, 256) = 57.73, *p* < .0001, $\eta_{p}^{2}$ = .18, indicating that when the advisee was proven to be right (M = 2.67, SE = .14) his or her dismissive behavior was judged as *less* arrogant than when he or she was proven to be wrong (M = 4.29, SE = .14). This result replicates the effect of Outcome found in Study 2.

The foregoing effect of Outcome was qualified by a significant Outcome x Expertise interaction, *F*(1, 256) = 3.93, *p* = .049, $\eta_{p}^{2}$ = .02. Additional *t*-tests conducted separately for each level of Outcome revealed that for the *negative* outcome the effect of Expertise was significant, *t*(130) = 2.32, *p* = .022, *d* = 0.4, indicating that the advisee’s dismissal was considered *more* arrogant when he or she had *lower* (M = 4.61, SE = .19) rather than *higher* expertise than the advisor (M = 3.90, SE = .21). No effect of Expertise was found for the positive outcome, *t* < 1 (Table 1). This result suggests that outcomes play a more important role in arrogance judgments than expertise.

**Table 1:** Ratings of the advisee’s arrogance.

	Cooking		Web Design		Total	
	Advisee's Expertise		Advisee's Expertise		Advisee's Expertise	
Outcome	Higher	Lower	Higher	Lower	Higher	Lower
**Negative**	3.71	4.39	4.06	4.79	3.90	4.61
	1.74	1.62	1.93	1.73	1.83	1.68
**Positive**	2.58	2.37	2.93	2.96	2.73	2.61
	1.61	1.24	1.41	1.49	1.53	1.36

*Note*. Table 1 summarizes Means and SDs of ratings of the advisee’s arrogance in Study 3a (1 –*not at all* to 7 –*very much*) by the outcome, by the advisee's relative expertise and by the vignettes.

Also, the effect of Type of relationships was significant, *F*(1, 256) = 4.39, *p* = .037, $\eta_{p}^{2}$ = .02, indicating that the advisee’s behavior was judged as more arrogant in the context of work (M = 3.69, SE = .15) than in the context of friendship (M = 3.27, SE = .14). We speculate that this effect reflects different levels of impact. While in the Cooking vignette, the advisee's dismissal of advice could have had harmful outcomes only for her, in the Web-design vignette it could have been harmful for the entire company. This effect might also stem from the advisee’s gender, since the advisee in the Cooking vignette was female and the advisee in the Web-design vignette was male. No other effects were significant, all *p*s > .2.

When Gender was added as a fourth factor to ANOVA, it was significant, *p* = .036, indicating that male (vs. female) participants rated the advisee as more arrogant (3.74 vs. 3.31). Also, the Gender x Outcome interaction was significant, indicating stronger effect of Outcome in female (M_neg_ = 4.29 vs. M_pos_ = 2.34) than in male participants (M_neg_ = 4.27 vs. M_pos_ = 3.20). Also, the Gender x Expertise interaction was marginally significant, *p* = .051, indicating a significant effect of Expertise in male participants (M_lower_ = 4.07 vs. M_higher_ = 3.04), *p* = .044, but not in female participants (M_lower_ = 2.69 vs. M_higher_ = 2.84). In addition, a Gender x Type of relationships x Outcome x Expertise 4-way interaction was marginally significant, *p* = .055. Inspecting this interaction showed that it stemmed from one anomaly: female participants rated the higher (vs. lower) expert advisee’s unsuccessful dismissal in the cooking vignette as more arrogant (4.30 vs. 3.72). Since these gender effects were not theoretically predicted and were not replicated in other studies, we refrain from interpreting them. Importantly, adding Gender to the ANOVA did not affect the significance or direction of the main findings.

#### Confidence judgments

An ANOVA with Type of Relationships, Outcome, and Advisee’s Relative Expertise as between participants’ factors was conducted on the ratings of the advisee’s arrogance. The results revealed a significant effect of Outcome, *F*(1, 256) = 22.94, *p* < .0001, $\eta_{p}^{2}$ = .08, indicating that when the advisee was proven to be right (M = 6.19, SE = .11) his or her dismissive behavior was judged as *more* confident than when he or she was proven to be wrong (M = 5.46, SE = .11).

Also, the effect of Type of relationships was significant, *F*(1, 256) = 10.56, *p* = .001, $\eta_{p}^{2}$ = .04, indicating that the advisee’s behavior was judged as more confident in the context of friendship (M = 6.08, SE = .11) than in the context of work (M = 5.58, SE = .11). This effect might stem from the assumption that people behave more confidently with their friends than with their colleagues; however, since we did not predict this effect, this interpretation should be taken with a grain of salt. Importantly, the main effect of Outcome worked differently for confidence judgments than for arrogance judgments—in fact, it even worked in the opposite direction. On top of that, neither the main effect of Expertise nor the Expertise x Outcome interaction effect on confidence was found significant, *p* > .19. Thus, the pattern of results for confidence judgments differed entirely from that of the arrogance judgments, supporting the discriminant validity of the arrogance measure (data in S5 Dataset.).

### Discussion

The results of Study 3a replicated the effect of outcome on arrogance judgments found in Study 2, lending further support to Hypothesis 2. In particular, dismissive behavior was judged as more arrogant when it was less justified by its outcome. In addition, the advisee’s dismissal of advice was judged as more arrogant when it was less justified by the advisee’s relative expertise, unless it was justified by the outcome of the dismissal. This pattern of results suggests a greater role of outcomes versus expertise in arrogance judgments. However, since there was no control condition (i.e., without outcome information) included, it is impossible to tell whether this conclusion extends to negative outcomes as well. We addressed this question in Study 3b.

## Study 3b: Outcomes vs. expertise (conceptual replication)

Across two cues of competence (expertise and outcome), two types of decisions (factual and hedonic), and two types of relationships (friendship and work), Studies 1-3a lent support to Hypotheses 1–3. However, since all three studies shared the same methodology of eliciting participants’ responses—arrogance ratings—they were vulnerable to the same criticism: the obtained results could be an artifact of participants having only one way to report their judgments, by tapping the more negative portion of the scale. In other words, it is possible that participants’ responses reflect their overall negative impressions of the protagonist rather than necessarily their specific attributions of arrogance. According to this critique, if participants had a choice, they might have applied different behavioral labels to the advisee’s behavior. For example, rejecting the advice of a more competent advisor could be interpreted as an unfriendly, antisocial behavior. However, we predicted that unjustified (vs. justified) dismissal of advice would be specifically identified as arrogance even when other labeling options were available.

Another goal of this study was to provide another test of Hypothesis 3. More specifically, we aimed to replicate our finding from Study 3a that *positive* outcome of advice dismissal moderates the effect of expertise, and to test whether negative outcome would also moderate the effect of expertise. To carry out the latter comparisons, we added a new condition wherein no outcome information was provided.

### Method

#### Participants

Two hundred and ten participants (111 females), ranging in age from 18 to 75 (Mean = 33.96, SD = 11.99), took part in the study on Amazon’s Mechanical Turk for 20 cents (USD) each.

#### Procedure and design

Participants were presented with the same robotics vignette used in Studies 1 & 2. The advisee’s relative expertise was manipulated in the same way as in Study 1 (though the equal competence condition of that study was not included here) and the outcome of the dismissal of advice was manipulated in the same way as in Study 2, yielding the following between participants’ design: 2 Advisee’s Relative Expertise (lower / higher than the advisor’s) x 3 Outcome (positive / negative / no information about outcome). After reading the vignette, participants were asked to choose one out of five words (*arrogant*, *confident*, *independent*, *unfriendly*, and *neutral*), which best described the advisee’s behavior. The order of the words was randomized for each participant.

### Results

There were no significant effects of gender, so they will not be discussed further.

First, in order to examine the frequency of “arrogant” versus “non-arrogant” responses, we recoded participants’ choices of “confident”, “independent”, “unfriendly” and “neutral” descriptors into one category of “non-arrogant” labels. The frequencies of all the descriptors are shown in Table 2.

**Table 2:** Labels assigned to the advisee’s behavior by outcome by the advisee’s expertise.

Outcome	Advisee’s Expertise	Arrogant	Confident	Independent	Neutral	Unfriendly	Total
**None**	**Lower**	71.4	14.3	11.4	2.9	0	100
	**Higher**	14.3	68.6	5.7	8.6	2.9	100
**Positive**	**Lower**	14.7	70.6	8.8	0	5.9	100
	**Higher**	8.6	74.3	8.6	5.7	2.9	100
**Negative**	**Lower**	69.4	13.9	8.3	8.3	0	100
	**Higher**	45.7	42.9	11.4	0	0	100

*Note*. Table 2 summarizes mean frequencies of behavioral labels (%) participants used to describe the advisee’s behavior in Study 3b by the outcome, by the advisee's relative expertise.

The first purpose of the analyses was to test the idea that *unjustified* dismissive behavior would be judged as more arrogant than *justified* behavior (Hypotheses 1 & 2). In order to do that, we recoded all conditions into two categories: “justified” and “unjustified” dismissals. Conditions wherein the advisee was more knowledgeable than the advisor, and either no outcome information was given, or the outcome of the dismissal of advice was positive, were coded as “justified”. Similarly, conditions where the advisee was less knowledgeable than the advisor, and either no outcome information was given, or the outcome of the dismissal of advice was negative, were recoded as “unjustified”. The two conditions in which the expertise information contradicted the outcome information were recoded according to Hypothesis 3, stating that outcome information is more important than expertise information. Thus, a condition wherein the advisee was less knowledgeable but proven to be right was coded as “justified”, and a condition wherein the advisee was more knowledgeable but proven to be wrong was coded as “unjustified”.

We then carried out a Chi Square test to examine whether advisee’s behavior was labeled as arrogant (vs. non-arrogant) more often in the “unjustified” (vs. “justified”) condition. The results confirmed our hypotheses, *χ*^2^(1) = 55.40, *p* < .001 (Munjustified = 62.26% vs. Mjustified = 12.50%). We also conducted binomial tests in each of the six conditions separately, comparing the observed percentage of “arrogant” classifications to the percentage expected by chance (20%). The results again confirmed Hypotheses 1 and 2, showing that in all “unjustified” conditions, the classification of the advisee’s behavior as arrogant was above chance, *p* < .001, while in all “justified” conditions it did not differ from chance, *p* > .12 (Table 2).

Our second goal in this study was to replicate the finding of Study 3a that outcome information is more important than expertise information. The analysis reported above already showed that in conditions in which outcome information contradicted expertise information, the advisee’s behavior was judged according to the outcome, but *not* according to expertise. To examine this issue more directly, we carried out a multinomial logistic regression on the percentage of “arrogant” responses with Advisee’s Expertise and Outcome as factors. The results revealed a significant effect of Advisee’s Expertise, *χ*^2^(1) = 11.91, *p* = .001 (Mlower = 52.38% and Mhigher = 22.86%), and a significant effect of Outcome, *χ*^2^(1) = 11.44, *p* = .001 (Mnegative = 57.75%, Mno outcome = 42.86%, Mpositive = 11.59%) in the predicted directions. More importantly, an Expertise x Outcome interaction was significant, *χ*^2^(2) = 6.64, *p* = .036.

To scrutinize the nature of this interaction, we carried out Chi Square tests on the effect of Outcome on each level of Expertise separately. Confirming the results of Study 3a, the effect of Outcome was found both when the advisee had lower expertise, *χ*^2^(2) = 15.88, *p* < .001, and higher expertise than the advisor *χ*^2^(2) = 28.64, *p* < .001.

Next, we carried out Chi Square tests on the effect of Expertise on each level of the Outcome variable separately. The effect of Expertise was significant in the predicted direction when no information regarding the outcome was given, *χ*^2^(1) = 23.33, *p* < .001 and when the outcome was negative, *χ*^2^(1) = 4.10, *p* = .043. Importantly, however, the effect of Expertise was not significant when the outcome was positive, *χ*^2^(1) = 0.63, *p* = .43. This pattern of results replicates and extends the results found in Study 3a, showing that outcome plays a more important role in perception of dismissive behavior than expertise. Indeed, the effect of outcome held regardless of the advisee’s relative expertise. In fact, a positive outcome eliminated the effect of expertise completely.

Including the “no outcome” condition in the design of this study allowed us to check whether a negative outcome would attenuate the effect of expertise as well. The multinomial logistic regression carried out with Expertise (lower / higher) and Outcome (negative outcome / no outcome information) on the percentage of “arrogant” responses showed that negative outcome (vs. no outcome information) attenuated the effect of expertise from 56.8% to 23.7%, *χ*^2^(1) = 4.96, *p* = .026 (data in S6 Dataset.).

### Discussion

The results of Study 3b confirmed Hypotheses 1 and 2, replicating the effects of expertise and outcome on a different dependent measure: behavior labeling. Another important contribution of this study was in replicating and extending the finding that outcome information has a stronger impact on arrogance judgments than expertise information. Two analyses corroborated this conclusion. First, in conditions where outcome information contradicted expertise information, the judgments were made according to the outcome information (i.e., “non-arrogant” when the outcome was positive and “arrogant” when the outcome was negative). Second, the interaction analyses showed that the effect of outcome was found regardless of the advisee’s level of expertise, whereas the effect of expertise was attenuated by negative outcomes and completely eliminated by positive outcomes. Taken together, these results confirm Hypothesis 3.

## Study 4: Manner vs. expertise

The primary goal of Study 4 was to examine the role of interpersonal manner in arrogance judgments. In order to do that, we manipulated the manner in which the protagonist dismissed the advice: polite, neutral, or rude. According to Hypothesis 4, the less polite manner should be perceived as more arrogant.

The second goal of this study was to explore the importance of interpersonal manner as compared to the advisee’s relative expertise. In order to carry out that comparison, we manipulated the *advisee's* relative expertise by presenting the advisor either as a novice or an expert. We expected to replicate the effect of expertise on arrogant judgments for normative (i.e., neutral and polite) manners. However, we predicted that a rude manner would be perceived as arrogant regardless of the advisee’s relative expertise (Hypothesis 5).

In addition, we wanted to extend our conclusions about the effect of the advisee’s relative expertise to more important decisions. In order to do that, we used a new vignette about a medical counseling situation in which advice was given about how to perform a liver transplant operation.

### Method

#### Participants

Two hundred and twenty three participants (121 females), ranging in age from 18 to above 40, took part in the study on Amazon’s Mechanical Turk for 20 cents (USD).

#### Procedure and design

In order to test our predictions, we presented participants with a medical counseling situation in which a surgeon dismissed the opinion of either the patient’s mother or of the chief surgeon. We also manipulated the manner of the dismissal, making it polite, neutral, or rude, yielding a 2 Advisee’s Relative Expertise (High / Low) x 3 Interpersonal Manner (polite / neutral / rude) between-participants design. Each participant was presented with one of the following versions of the vignette:

Alex is a surgeon meeting with a patient and **her mom** / **a chief surgeon** before a liver transplant operation. Alex is showing on the x-ray the points of incision, where the doctors will operate. The **patient’s mom** / **chief surgeon** suggests to make the incision lines less crooked. Alex says: **“I can see why you are saying that, but I still think the incision lines are fine.” / "I think the incision lines are fine.” /** “**You don't know what you are talking about. I think the incision lines are fine.”**

The participants judged the extent to which the advisee’s behavior was arrogant, and the extent to which the both actors were polite, knowledgeable, and confident (1 –*not at all*, to 7 –*very much*). Perceived politeness and knowledge were measured as manipulation checks, and perceived confidence was measured for establishing the discriminant validity of the arrogance measure.

### Results

There were no significant effects of gender, so it will not be discussed further.

#### Manipulation checks

Because of a technical problem with the software, 13 responses on the manipulation checks were not collected, so these analyses refer to only 210 participants. First, we checked whether the manipulation of the advisee’s manner was effective. An ANOVA with Manner (polite / neutral / impolite) as a between-participants factor conducted on the advisee’s politeness ratings was significant, *F*(1, 207) = 30.64, *p* < .0001, $\eta_{p}^{2}$ = .23. Planned contrasts showed that the rude manner was rated as less polite (M = 2.28) than the neutral manner (M = 3.68), *p* < .0001 and the neutral manner was rated as less polite than the polite manner (M = 4.40), *p* = .006. Thus, our manipulations of the advisee’s manner were effective.

Then, we checked whether the manipulation of expertise affected participants’ ratings of the advisee’s perceived competence. The results showed that the advisee was judged as *less* knowledgeable when he was advised by the chief surgeon (M = 4.55), than when he was advised by the patient’s mother (M = 5.80), *t*(208) = 5.98, *p* < .0001, confirming that our manipulations of the advisee’s relative expertise were effective. Not surprisingly, analyses of the advisor’s perceived competence showed that the more expert advisor was perceived as more knowledgeable (M = 6.07) than the less expert advisor (M = 2.67), *t*(208) = 19.12, *p* < .0001.

#### Arrogance judgments

In order to investigate how arrogance judgments were influenced by the advisee's relative expertise and his/her manner, we performed an ANOVA with these two between-participants factors. The results revealed a significant effect of the Advisee's Relative Expertise, *F*(1, 217) = 24.15, *p* < .0001, $\eta_{p}^{2}$ = .1, indicating that when the advisee’s expertise was *lower* than that of the advisor, the advisee’s behavior was judged as *more* arrogant (M = 5.1, SE = .16) than when his/her expertise was *higher* than that of the advisor (M = 4.00, SE = .16). The effect of Manner was also significant, *F*(1, 217) = 3.84, *p* < .0001, $\eta_{p}^{2}$ = .17. Planned contrasts showed that when the advisee’s manner was rude, it was judged as *more* arrogant (M = 5.61, SE = .19) than when it was either neutral (M = 4.06, SE = .19) or polite (M = 3.99, SE = .19), *p* < .0001; the difference between the neutral and polite forms was not significant, *p* > .7.

**Fig 1:**
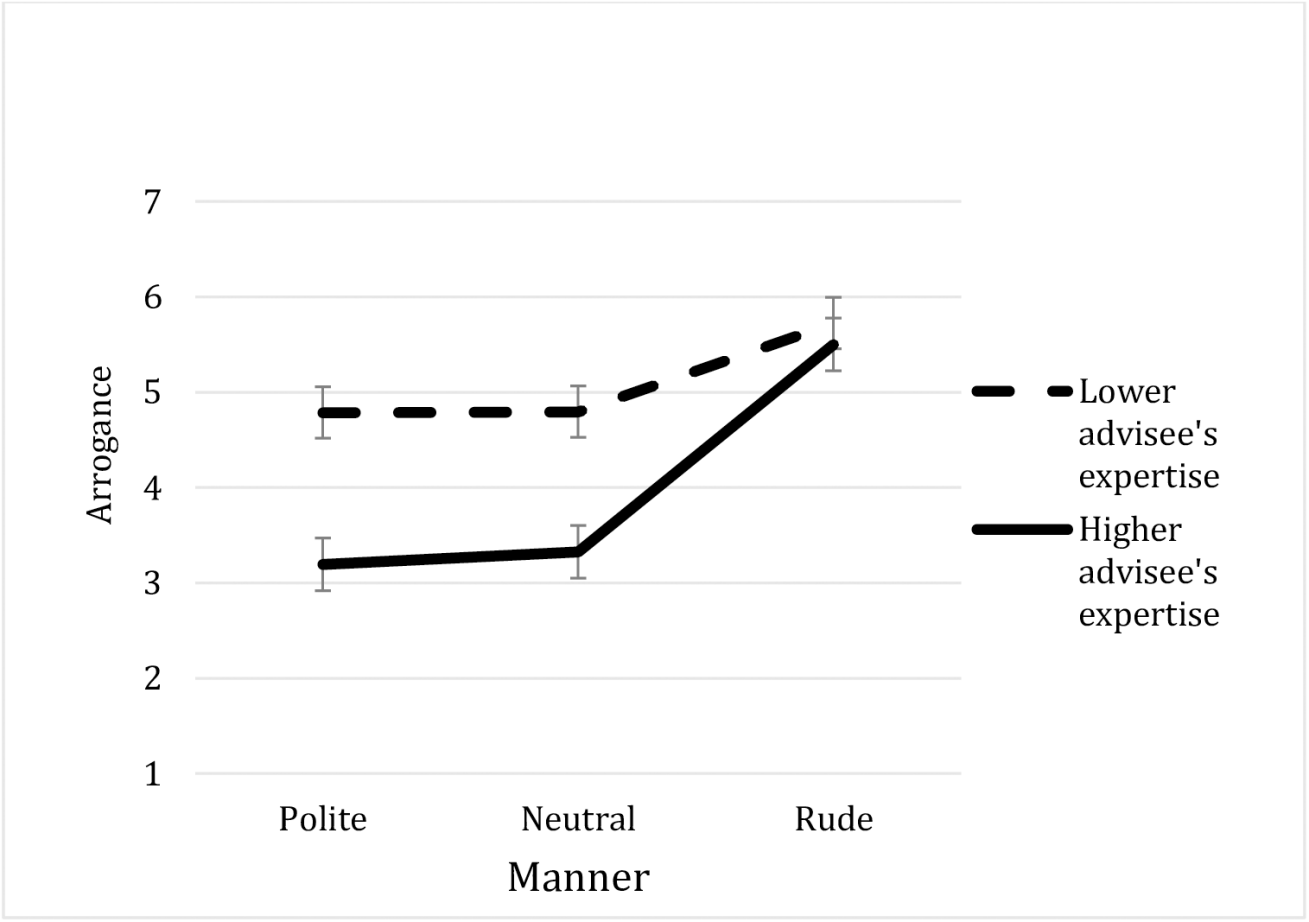
Ratings of the advisee's arrogance by his/her relative expertise by his/her manner (Study 4).

More importantly, the Advisee's Relative Expertise x Manner interaction was significant, *F*(1, 217) = 3.84, *p* = .023, $\eta_{p}^{2}$ = .03 (Fig 1). Additional *t*-tests run for each level of the Manner factor separately revealed the significant effect of the Advisee's Relative Expertise both for the neutral (M_low_ = 4.79 vs. M_high_ = 3.32), *t*(74) = 3.73, *p* < .0001, *d* = .85 and for the polite manners (M_low_ = 4.78 vs. M_high_ = 3.19), *t*(71) = 4.03, *p* < .0001, *d* = .94, but not for the rude manner (M_low_ = 5.72 vs. M_high_ = 5.5), *t*(72) < 1, *d* = .14.

We also conducted ANOVAs for each level of the Advisee's Relative Expertise separately. Both when the advisee’s relative expertise was *lower* and when it was *higher*, the effect of Manner was significant, *F*(1, 108) = 20.87, *p* < .0001, $\eta_{p}^{2}$ = .28 and *F*(1, 109) = 4.23, *p* = .02, $\eta_{p}^{2}$ = .07, respectively. For both levels of the Advisee's Relative Expertise, the planned contrasts showed that the arrogance judgments were higher for the rude than for the neutral and the polite manners of behavior, *p* < .01 and there was no difference between the last two manners, *p* > .7.

### Confidence judgments

In order to investigate how confidence judgments of the advisee were influenced by the advisee's relative expertise and her manner, we performed an ANOVA with these two between-participants factors. No significant effects were found, *p* > .13. The lack of effect of manner and expertise manipulations on the perceived advisee’s confidence supports the discriminant validity of the arrogance measure. An ANOVA with the same factors conducted on the perceived advisor’s confidence showed, not surprisingly, that the more expert advisor was perceived as more confident (M = 6.24, SE = .12) than the less expert advisor (M = 4.72, SE = .13), *F*(1, 204) = 69.15, *p* < .001, $\eta_{p}^{2}$ = .25 (data in S7 Dataset.).

### Discussion

The results of Study 4 once again offered support for Hypothesis 1, replicating the effect of expertise on arrogance judgments. They also showed that the manner in which people dismiss others’ opinions is crucial to those judgments. Both when the advisee had lower and higher relative expertise, her dismissal of the advice was perceived as more arrogant when it was expressed in a rude way than when it was expressed in a normative way. The results also supported Hypothesis 4, showing that rude (vs. normative) dismissal of advice make an advisee appear more arrogant. Of greater interest, supporting Hypothesis 5, interpersonal considerations overrode competence considerations: when an advisee dismissed advice in a rude manner, he or she was perceived as equally arrogant, regardless of his or her relative expertise.

These results suggest that dismissive behavior is primarily an interpersonal rather than an intellectual phenomenon. That is, while in subtle cases of dismissive behavior people use the advisee’s greater expertise to infer that he or she dismissed advice because of his or her better grasp of the truth, a rude dismissal leaves no room for a doubt that it was driven by arrogance. Yet, it is premature to conclude that interpersonal considerations influence the perception of dismissive behavior more than competence ones, because so far we have compared manner to only one cue of competence (expertise). Indeed, expertise indicates one’s competence across many cases, but does not necessary indicate one’s correctness (or fault) in the specific case of disagreement. So, it was possible that manner trumped expertise because it was perceived as a more direct cue of arrogance. That is why we decided to compare the effect of the interpersonal manner to the effect of a more direct cue of the advisee’s correctness: the outcome of the dismissal.

## Study 5: Manner vs. outcomes

In Study 4, we found support for our Hypothesis 5, which states that interpersonal considerations trump competence considerations. The primary goal of Study 5 was to extend this finding to another cue of competence: the outcome of the dismissal. As in Study 4, we expected to replicate the effect of outcome for normative manners. However, we predicted that a rude dismissal of advice would be perceived as arrogant regardless of the outcome of the dismissal (Hypothesis 6). A secondary goal of this study was to replicate the effect of interpersonal manner on arrogance judgments (Hypothesis 4) in the context of less important decisions regarding matters of taste.

### Method

#### Participants

Two hundred eighty four participants (146 males, one participant did not indicate his or her gender), ranging in age from 18 to above 40, took part in the study on Amazon’s Mechanical Turk for 20 cents (USD) each.

#### Procedure and design

We presented participants with a new vignette in which a designer in an advertising company dismissed his colleague’s advice about the logo design on a shampoo advertisement. The outcome of the advisee’s decision to dismiss the advice was manipulated to be either positive (the pilot of the ad showed that people remembered the logo pretty well) or negative (the pilot showed that people did not remember the logo). We also manipulated the manner of the dismissal, rendering it polite, neutral, or rude. These manipulations yielded a 2 Outcome (positive / negative) x 3 Manner (polite / neutral / rude) between-participants design. Accordingly, each participant was presented with one of the 6 versions of the vignette:

Taylor is a designer in an advertising company and is working on a new ad for shampoo. While Taylor is working on the ad, Jamie, a colleague who works at the same the company, enters the room. Jamie looks at the draft of Taylor’s ad and suggests that the logo should be more prominent, otherwise people will not pay attention to it. Taylor says: "**I see what you mean and you might be right, but I still think it looks good." / "I think it looks good." / "You have no idea what you’re talking about. It looks good."**
A week later, the pilot of Taylor’s ad showed that people **remembered the logo pretty well** / **did not remember the logo.**

Participants judged the extent of the advisee’s arrogance, politeness, and knowledge (1 –*not at all*, to 7 –*very much*). The last two questions served as manipulation checks. Finally, participants answered a catch question that tested their comprehension of the story. We added this question to make sure that participants understood which of the actors (the advisee or the advisor) turned out to be right, since this understanding was deemed to be critical for the arrogance judgments.

### Results

First, we checked the accuracy of the participants' answers on the catch question. Data from 33 participants was discarded from further analyses because they did not answer it correctly. Thus, data of 251 participants (127 males and 123 females; one participant did not report gender) remained for analysis.

There were no significant effects of gender, so they will not be discussed further.

#### Manipulation checks

First, we checked whether the manipulation of the advisee’s manner was effective. An ANOVA with Manner (polite / neutral / rude) as a between-participants factor conducted on the advisee’s politeness ratings was significant, *F*(1, 248) = 99.70, *p* < .0001, $\eta_{p}^{2}$ = .45. As expected, planned contrasts showed that the rude manner was rated as less polite (M = 2.62) than the neutral manner (M = 4.52) and the neutral manner was rated as less polite than the polite manner (M = 5.45), *p* < .0001 for both comparisons.

We then checked whether the manipulation of outcome affected participants’ ratings of the advisee’s competence. The results showed that when the advisee turned out to be right he was judged as more knowledgeable (M = 5.59) than when he turned out to be wrong (M = 4.34), *t*(249) = 8.92, *p* < .0001, *d* = 1.13. Thus, the manipulation of the outcome changed the perceived competence of the advisee, as we intended.

#### Arrogance judgments

In order to check how arrogance judgments were influenced by outcome and the manner, an ANOVA with these two factors was conducted on the arrogance ratings of the advisee. The results showed a significant effect of Outcome, *F*(1, 245) = 35.35, *p* < .0001, $\eta_{p}^{2}$ = .13, indicating that when the advisee turned out to be right, his or her behavior was judged as less arrogant (M = 3.51), than when he turned out to be wrong (M = 4.63). Also, the effect of Manner was significant, *F*(1, 245) = 46.33, *p* < .0001, $\eta_{p}^{2}$ = .27. Planned contrasts showed that when the advisee’s behavior was rude, it was judged as more arrogant (M = 5.33) than when it was either neutral (M = 3.64) or polite (M = 3.23), *p* < .0001; the difference between the neutral and polite forms of behavior was marginally significant, *p* = .08. Thus, both the effect of outcome and the effect of manner on arrogance judgments were replicated.

**Fig 2:**
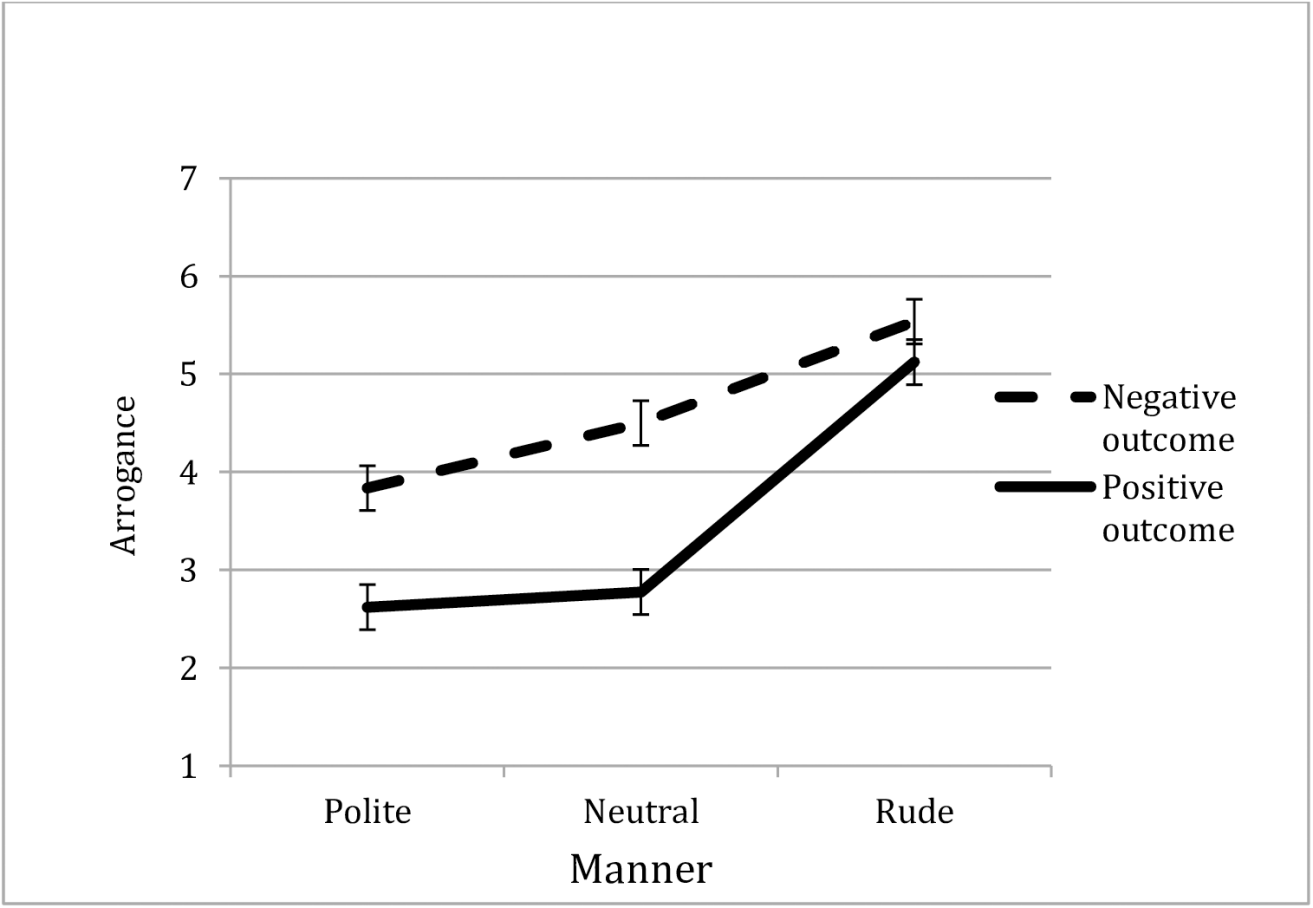
Ratings of the advisee's arrogance by the outcome and the manner of his/her behavior (Study 5).

More importantly, the Outcome x Manner interaction was significant, *F*(1, 245) = 4.07, *p* = .018, $\eta_{p}^{2}$ = .03 (Fig 2). Additional *t*-tests run for each level of the Manner factor separately revealed that in the spectrum of normative behavior, when the advisee turned out to be right (i.e., where the outcome was positive), his/her behavior was judged as less arrogant than when [s]he turned out to be wrong (i.e., negative outcome), (M_positive_ = 2.78 vs. M_negative_ = 4.50), *t*(82) = 5.76, *p* < .0001, *d* = 1.26, and (M_positive_ = 2.62 vs. M_negative_ = 3.84), *t*(83) = 3.51, *p* = .001, *d* = 0.76, for neutral and polite behavior respectively. However, for the rude manner of dismissal, there was no significant difference between the positive (M = 5.12) and the negative outcomes (M = 5.54), *t*(80) = 1.26, *p* = .21, *d* = 0.28.

ANOVAs conducted for each outcome separately revealed significant effects of Manner both in the case of positive *F*(1, 120) = 39.50, *p* < .0001, $\eta_{p}^{2}$ = .40 and negative outcomes, *F*(1, 125) = 12.84, *p* < .0001, $\eta_{p}^{2}$ = .17. Planned contrasts showed slightly different results for each outcome. Namely, when the outcome was *positive*, the rude dismissal was judged as more arrogant than the normative ones, *p* < .0001; there was no difference between the neutral and polite behaviors, *p =* .62. Similarly, when the outcome was *negative*, the rude dismissal was judged as more arrogant than normative dismissal, *p* < .0001; however, the neutral manner was judged as more arrogant than the polite one, *p* = .048, suggesting that negative outcomes make the differences in politeness loom larger (data in S8 Dataset).

### Discussion

The results of Study 5 confirmed Hypotheses 2 and 4, replicating the effects of outcome and manner on arrogance judgments that we found in the previous studies. In addition, consistent with the findings of Study 4, interpersonal considerations overrode the influence of the competence considerations (Hypothesis 6). Specifically, when the manner of dismissal was rude, it was perceived equally as more arrogant, regardless of whether the advisee turned out to be right or not.

So far, we found evidence in support of Hypotheses 1–6, indicating that both an advisee’s relative competence (indicated by his/her expertise and/or the outcome of his/her dismissal of advice) and manner determine whether he or she would be perceived as arrogant. Moreover, we showed that the outcome of the dismissal trumps expertise, and the manner of the dismissal trumps both expertise and outcomes. But how surprising are these findings compared to what people naively think about arrogance? In order to answer this question, we conducted two additional studies.

## Study 6a: Naïve theories about arrogance

The goal of Study 6a was to explore people's lay beliefs about the importance of manner, expertise, and outcomes in arrogance judgments. In order to do that, we presented participants with the cooking vignette used in Studies 2 and 3a while omitting details about the advisee's relative expertise, manner, and the outcomes of his or her dismissal.

### Method

#### Participants

Fifty participants (27 males), ranging in age from 18 to above 40, took part in the study on Amazon’s Mechanical Turk for 20 cents (USD).

#### Procedure and design

We presented participants with the following vignette in which an advisee dismissed his or her friend's advice about cooking:

Casey has just entered a pastry contest in the community. Casey is preparing a cake at home when Jordan comes to visit. Jordan tastes the dough and advises Casey to add more sugar to it. Casey refuses.

We asked participants to rate the extent (1—*not at all* to 7—*very much*) to which they would like to receive additional information about the *manner* of Casey's behavior, his or her relative *expertise*, and the *outcome* of his or her refusal, in order to decide whether the behavior was arrogant. The order of these three types of information was determined randomly for each participant.

### Results

There were no significant effects of gender, so it will not be discussed further. ANOVA with Manner, Expertise and Outcome ratings as repeated measures revealed a significant difference in importance between the three types of information, *F*(2, 98) = 10.34, *p* < .0001, $\eta_{p}^{2}$ = .17. Planned contrasts showed that Manner (M = 5.44, SE = .23) and Expertise (M = 5.1, SE = .23) were rated as more important than Outcome (M = 3.8, SE = .29), *p* < .001; no difference was found between the first two types of information, *p* > .3 (data in S9 Dataset.).

### Discussion

In contrast to how people actually use expertise and outcomes information in their arrogance judgments (Studies 3a and 3b), Study 6a showed that people consider expertise to be more important than outcomes. Also, in contrast to Study 4, which demonstrated that interpersonal manner trumps expertise, Study 6a showed that people think that manner and expertise are equally important for arrogance judgments. Taken together, these results suggest that people overestimate the effect of expertise and underestimate the effect of outcomes on arrogance judgments. How general is this misperception of the hierarchy of arrogance cues? We tried to answer this question in the next study.

## Study 6b: Naive theories (conceptual replication)

Study 6b was conducted in order to replicate the findings of Study 6a in a different context. In Study 6a, the vignette described a conversation between two friends about a hobby that one of them pursued. In such a situation, only the advisee could suffer from the potential negative outcomes of his or her dismissive behavior. It is possible that outcome information will be of lesser importance than expertise in this context within friends; whereas in the context of work, where the outcomes of a colleague's dismissal might be harmful for a company, their relative importance might be reversed. It is also possible that the relative importance of manner and expertise considerations would vary in a different context. To test whether the pattern of results obtained in Study 6a generalized beyond specific situations, we asked participants to rate the importance of the arrogance cues in the ad design vignette used in Study 5.

### Method

#### Participants

Fifty-one participants (28 males), ranging in age from 18 to above 40, took part in the study on Amazon’s Mechanical Turk for 20 cents (USD).

#### Procedure and design

Participants were presented with a vignette in which a designer dismissed his or her colleague’s advice about improving the logo on a shampoo advertisement:

Taylor is a designer in an advertising company and is working on a new ad for shampoo. While Taylor is working on the ad, Jamie, a colleague who works at the same company, enters the room. Jamie looks at the draft of Taylor’s ad and suggests that the logo should be more prominent, otherwise people will not pay attention to it. Taylor refuses.

Participants were asked to rate the importance of the same types of information as used in Study 6a. The order of these three types of information was determined randomly for each participant.

### Results

There were no significant effects of gender, so they will not be discussed further. An ANOVA with Manner, Expertise, and Outcome ratings as repeated measures, revealed again a significant difference in importance between the three types of information, *F*(2, 100) = 9.09, *p* < .0001, $\eta_{p}^{2}$ = .15. Planned contrasts showed that Manner (M = 5.86, SE = .19) and Expertise (M = 5.53, SE = .20) were rated as more important than Outcome (M = 4.53, SE = .28), *p* < .001; also, replicating Study 6a, no difference was obtained between the first two types of information, *p* > .24 (data in S10 Dataset.).

### Discussion

Results of Study 6b showed that in the context of work, participants also considered information concerning manner and expertise to be equally important for arrogance judgments, and more important than outcome information. Thus, the results of Study 6b entirely replicated the pattern of results obtained in Study 6a. Taken together, Studies 6a and 6b demonstrate that people's naïve theories about the relative importance of arrogance cues differ from their actual relative importance, as shown in Studies 1–5.

## General discussion

The main goal of this paper was to investigate when a person who dismisses another individual’s advice is perceived as arrogant. Based on previous literature on person perception [23–26] and the perception of arrogance [27–30], we hypothesized that people may use both competence and interpersonal manner information to infer an advisee’s arrogance. More specifically, we predicted that an advisee who was less competent than the advisor would appear more arrogant. The results of Study 1 supported this prediction when the advisee’s competence was indicated by his or her expertise, and Study 2 replicated it with a different cue of competence: the outcome of the advice dismissal. Studies 3a and 3b showed that outcome information trumped expertise information across two different types of responses (i.e., rating and choice) and two different interpersonal contexts (i.e., friendship and work relations). That is, the effect of expertise on arrogance judgments was attenuated by negative outcome and completely eliminated by positive outcome information.

We also hypothesized that the manner in which people dismiss advice would influence their perceived arrogance more than their competence. The results of Studies 4 and 5 offered support for this hypothesis, showing that a rude dismissal of advice was perceived as more arrogant than a normative (either polite or neutral) dismissal, regardless of the advisee’s expertise and the outcome of the decision. Yet, the effects of expertise and outcomes were obtained only when the manner was normative, but not when it was rude; in the latter case, the advisee was perceived as equally arrogant, regardless of his or her expertise and the outcome of his or her decision to dismiss the advice. Thus, our results suggest that arrogance is primarily an interpersonal rather than an intellectual phenomenon [33].

Our findings also provide insight into people’s naïve theories of arrogance. In particular, whereas people expect arrogance judgments to be more influenced by expertise than by outcome information, actual arrogance judgments are swayed more by outcome information. In addition, people mistakenly believe that expertise and manner are equally important for arrogance judgments. This misconception might explain, in part, why people behave arrogantly even though they value modesty[3,4]. It may happen because they think that their superiority in competence will compensate for their rude manner.

### Arrogance vs. related constructs

#### Arrogance

Our findings shed light on the nature of arrogance and help distinguish it from other related constructs. In particular, in the aforementioned studies of Hareli [27,28], an arrogant person was depicted as a successful individual who attributed his success to his ability. In contrast, Johnson’s study suggested that arrogant employees are the less productive and less smart individuals. While these portraits of arrogant people might seem contradictory at first glance, we think that they can be reconciled in light of our findings. Indeed, our results show that both competence and interpersonal manner influence the perception of arrogance. So it is possible that successful and unsuccessful people are judged as arrogant for different reasons. Successful people might be perceived as arrogant because their manner violates some norms of interpersonal behavior (e.g., the norm of being modest or the norm of being polite). Unsuccessful people may be perceived as arrogant for a different reason—because their dismissal of others’ opinions is not justified by their competence.

#### Arrogance vs. hubristic pride

Our research also contributes to the research on *hubristic pride*. Tracy and Robins [62] define hubristic pride as one of the facets of pride, along with authentic pride. Both hubristic and authentic prides are defined as positive self-conscious emotions that are elicited by achievements; they differ in the factors to which people attribute the success. In the case of authentic pride, people attribute the success to internal, unstable, and controllable factors such as effort, while in the case of hubristic pride, people attribute the success to internal, stable, and uncontrollable factors such as ability [63,64]. This definition of hubristic pride is quite similar to the conceptualization of arrogance suggested by Hareli and his colleagues [27,28]. Although our results show that arrogance is a broader phenomenon that includes dismissive behaviors as well (in fact, people associate arrogant behavior more often with dismissive than with boastful behavior), Tracy and Robbins’ [64] concept of *trait hubristic pride* might be tantamount to arrogance. Indeed, to measure *trait hubristic pride* they used such items such as “arrogant,” “conceited,” and “smug”.

#### Arrogance vs. contempt

Similar to hubristic pride, contempt is another emotion that may be associated with arrogance. Indeed, contemptuous behavior can be similar to arrogant behavior in manner (e.g. ignoring another person’s suggestions) [65] [65]. Note, however, that contempt differs from arrogance in that contempt is an emotion [66–68], while arrogance usually refers to an attitude, behavior or trait [6,27]. Moreover, conempt is usually elicited when another person’s behavior violates some interpersonal standards (e.g., immoral, unethical, or otherwise unsavory behavior)[69], while arrogance is typically not a reaction to another person's unacceptable behavior.

#### Arrogance vs. overconfidence

Overconfidence is different from arrogance in its scope, causes, and manifestations. Regarding its scope, overconfidence refers to task abilities while arrogance refers to more holistic judgments of the self and its value relative to others’ value. In addition, the psychological underpinnings of overconfidence may differ from those of arrogance. In our view, while arrogance is driven by an *inter*personal motivation to be better than other people, overconfidence is an *intra*personal phenomenon driven by the cognitive evaluation of one’s own ability based on task performance or task difficulty [70,71]. In addition, on the level of behavior, arrogance is directed (explicitly or implicitly) at other people, such as disparaging others and/or dismissing their opinions, while overconfident behavior typically refers to one’s beliefs about his own ability or chances of achieving a goal.

#### Arrogance vs. stubbornness

Similar to arrogance, stubbornness might also lead to an unwillingness to change one’s opinions, but for different reasons. Stubbornness is driven by the epistemic motivation of resistance to change[72], while arrogance is driven by social motivations such as self- and/or status-enhancement[73,74]. Thus, we predict that perceptions of stubbornness (vs. arrogance) will be less influenced by the manner of dismissal.

#### Arrogance vs. narcissism

Narcissism is a complex psychological construct that has been studied in clinical and social psychology as a stable disposition. According to the clinical definition of narcissism (DSM-IV), arrogant behavior is only one of the nine symptoms of narcissistic disorder (others symptoms include envy, need for admiration, the tendency to exploit others, etc.). In social psychology, narcissism has been studied as a normal trait. The most common tool used to measure narcissism is Narcissistic Personality Inventory[75]. This inventory consists of seven components, among which only three—superiority, entitlement and vanity—positively correlate with trait arrogance [6]. Thus, narcissism is a related but different psychological construct. Moreover, since research on narcissism has predominantly focused on the self-regulation processes of narcissistic personalities[76], it has shed little light on the determinants of perceptions of arrogance and on the situational determinants of arrogant behavior—a gap that our studies have aimed to fill.

### Social judgments

Our results show that when it comes to arrogance judgments, social concerns are more important than epistemic concerns. This finding adds to similar findings about communion and agency dimensions in the area of person perception [24,26,55]and to findings about warmth and competence dimensions in the area of group perception[25]. Wojciszke and his colleagues [26] talk about communion traits serving the promotion of others’ interests, as opposed to agency traits, which serve the promotion of self-interest. They argue that when evaluating other people, communion traits play a more important role than agency traits. While in their studies, the relative importance of the communion and agentic traits was inferred from abstract judgments, in our studies, participants were presented with specific behaviors of the advisee in which communion (interpersonal manner) and agency (competence) concerns were pitted against each other. In accordance with Wojciszke and colleagues’ theory [26], we found that communion concerns trumped agency concerns in this context as well.

### Limitations and future directions

Although in our studies we tried to present participants with typical social situations representing the varied contexts of human relationships, the external validity of our conclusions is obviously limited by the methodology we used. In particular, we used vignettes to describe the advisee’s dismissive behavior. This method was appropriate for our research because it allowed us to highlight the main information we were interested in (i.e., manner, expertise, and outcome) and to manipulate it in an experimentally rigorous way. Vignettes have frequently been used in past research on perception of arrogance [23,24,26] and hubristic pride[30,77]. Yet, further research is needed to explore how people make arrogance judgments in reference to real behavior.

Another potential limitation of our studies is the fact that we used only one item to measure perceived arrogance. We chose to do so because this item had the best face validity for measuring participants’ perception of advisee’s behavior, and we worried that additional items might just add unnecessary noise. While some readers might be concerned about the low reliability of this dependent measure, note that, if anything, its low reliability would have decreased our chances of obtaining any effects. However, in spite of this, our effects were found to be significant and consistent. Having said that, a multiple-item scale may be a better measure for assessing the arrogance of various behaviors in different contexts [22].

Another feature of our methodology was the fact that the data was collected online rather than in a well-controlled lab environment. Note, however, that we found significant and consistent results despite the potentially greater noise of online studies. Indeed, a growing number of papers have shown that data obtained on MTurk are at least as reliable as those obtained via traditional methods[78,79]. Another potential problem for MTurk studies is repeated participation of the same participants. In order to prevent repeated participation, we collected participants’ MTurk IDs and screened out those who had already taken any of our studies. The third potential problem with the online studies is biased responding (e.g., always choosing the same option on a scale or making overly harsh judgments). Note, however, that biased responding is unlikely to yield significant differences in the between-participants’ designs that were used in our studies. Moreover, we also used catch questions to verify that participants followed the instructions properly. The advantage of online platforms, on the other hand, is that they offer researchers access to a more diverse population as compared to those of lab studies, which are usually limited to psychology students. In particular, in our studies the participants were from 52 countries (though the majority of participants were from the US) and varied in age from 18 and 79.

In our studies, we focused only on one type of arrogant behavior: dismissal of advice. Future studies should test our conclusions in the context of other types of arrogant behavior, such as communicating success. One interesting question for future research is how boasting and dismissive behavior relate to each other. One possibility is that both behaviors are substitutable means to the same goal[80]. While boasting serves this goal by putting one up, dismissive behavior does it by putting others down. Alternatively, it is possible that boasting and dismissive behaviors serve different goals. For example, boasting serves the goal of self-enhancement[74], while dismissive behavior serves the goal of establishing social status[73]. In any case, it is important to explore the motivations that drive boasting and dismissive behaviors, as well as situational and dispositional factors that moderate those behaviors.

### Practical implications

Understanding perceptions of arrogance has a number of possible practical implications. Doctors, lawyers, professors, and other experts in their fields often encounter situations in which their opinions are challenged by less knowledgeable others (e.g., patients, clients, or students). Such behavior of less knowledgeable people might be annoying, and experts might be tempted to dismiss them outright, sometimes in an impatient or even a rude manner. Although experts might believe that their behavior is justified by their knowledge, our findings show that it will still be perceived as arrogant, creating a negative impression of the dismissive individual.

This conclusion parallels findings in the literature on the “fair process effect.” This literature shows that perceived procedural fairness positively affects how people react to the outcomes they receive from authorities [81]. People may be dissatisfied with the same outcome, if they think that they were not treated with dignity by the person who made a decision[82]. Thus we (humbly!) recommend that individuals refrain from deviating from normative behavior, even when such a deviation seems to be justified by one’s greater knowledge or other forms of superiority.

### Conclusions

In summary, our results show that two factors influence whether the dismissal of advice will be perceived as arrogant: an advisee’s relative competence, and the manner of the advice dismissal. Two cues of competence can justify people’s dismissive behavior: their relative expertise and the outcomes of their dismissal. Contrary to what people think, outcomes are more important than expertise. Also, although people believe that manner and expertise are equally important for arrogance judgments, our findings show that rude behavior is not excused by one's superior competence.

## Supporting information

S1 Dataset Dataset of pilot 1. (SAV)

S2 Dataset Dataset of pilot 2. (SAV)

S3 Dataset Dataset of study 1. (SAV)

S4 Dataset Dataset of study 2. (SAV)

S5 Dataset Dataset of study 3a. (SAV)

S6 Dataset Dataset of study 3b. (SAV)

S7 Dataset Dataset of study 4. (SAV)

S8 Dataset Dataset of study 5. (SAV)

S9 Dataset Dataset of study 6a. (SAV)

S10 Dataset Dataset of study 6b. (SAV)
